# Direct on-the-spot detection of SARS-CoV-2 in patients

**DOI:** 10.1177/1535370220941819

**Published:** 2020-07-16

**Authors:** Nadav Ben-Assa, Rawi Naddaf, Tal Gefen, Tal Capucha, Haitham Hajjo, Noa Mandelbaum, Lilach Elbaum, Peter Rogov, Daniel A King, Shai Kaplan, Assaf Rotem, Michal Chowers, Moran Szwarcwort-Cohen, Mical Paul, Naama Geva-Zatorsky

**Affiliations:** 1 Department of Cell Biology and Cancer Science, Technion Integrated Cancer Center (TICC),Rappaport Faculty of Medicine, Technion-Institute of Technology, Haifa 3525433, Israel; 2 Department of Oral and Maxillofacial Surgery, Rambam Medical Care Center, Haifa 31999, Israel; 3 Department of Immunology, Rappaport Faculty of Medicine, Technion-Israel Institute of Technology, Haifa 3525433, Israel; 4 Department of Neuroscience, Rappaport Faculty of Medicine, Technion-Israel Institute of Technology, Haifa 3525433, Israel; 5 Independent Researcher, Winchester, MA 01890, USA; 6 Pulmonary and Respiratory Critical Care Division, Meir Medical Center, Kfar Saba 44281, Israel; 7 Robiotec Ltd, Rehovot 7634709, Israel; 8 Independent Reearcher, Newton, MA 02459, USA; 9 Infectious Diseases Unit, Meir Medical Center, Kfar Saba 44281, Israel; 10 Sackler Faculty of Medicine, Tel-Aviv University, Tel-Aviv Isarel, 3200; 11 Virology Laboratory, Rambam Health Care Campus, Haifa 31999, Israel; 12 Division of Infectious Diseases, Rambam Health Care Center, and Rappaport Faculty of Medicine, Technion-Israel Institute of Technology, Haifa 31999, Israel; 13 MaRS Centre, Canadian Institute for Advanced Research (CIFAR) Azrieli Global Scholar, Toronto, ON M5G 1M1, Canada

**Keywords:** Molecular, Covid-19, SARS-CoV-2, RT-LAMP, surveillance, pandemic

## Abstract

Many countries are currently in a state of lockdown due to the SARS-CoV-2 pandemic. One key requirement to safely transition out of lockdown is the continuous testing of the population to identify infected subjects. Currently, detection is performed at points of care using quantitative reverse-transcription PCR, thus requiring dedicated professionals and equipment. Here, we developed a protocol based on reverse transcribed loop-mediated isothermal amplification for the detection of SARS-CoV-2. This protocol is applied directly to SARS-CoV-2 nose and throat swabs, with no RNA purification step required. We tested this protocol on over 180 suspected patients, and compared the results to those obtained using the standard method. We further succeeded in applying the protocol to self-collected saliva samples from confirmed cases. Since the proposed protocol can detect SARS-CoV-2 from saliva and provides on-the-spot results, it allows simple and continuous surveillance of the community.

**Impact statement:**

Humanity is currently experiencing a global pandemic with devastating implications on human health and the economy. Most countries are gradually exiting their lockdown state. We are currently lacking rapid and simple viral detections, especially methods that can be performed in the household. Here, we applied RT-LAMP directly on human clinical swabs and self-collected saliva samples. We adjusted the method to allow simple and rapid viral detection, with no RNA purification steps. By testing our method on over 180 human samples, we determined its sensitivity, and by applying it to other viruses, we determined its specificity. We believe this method has a promising potential to be applied world-wide as a simple and cheap surveillance test for SARS-CoV-2.

## Introduction

During a pandemic, surveillance is crucial for minimizing viral spread. The common and approved detection method used worldwide requires professional experience in sampling, carrying out the reaction and analyzing the results. Moreover, it requires dedicated machines and chemical reagents, as well as sophisticated sample collection and transport logistics. Due to these labor-intensive and cumbersome requirements, the number of detection tests performed per day is limited. As such, many patients in the community are not sampled, let alone sampled frequently. Such limited surveillance necessitates global and strict quarantine requirements, which threaten the global economy.

Given that detection is key, a simple and easy detection method, preferably one that can be performed and interpreted on-the-spot, could help relieve current limitations, and thus contribute to a safe and efficient lockdown exit strategy. Accordingly, rapid and simple serological tests that can, in principle, be conducted at home are being developed.^1^ However, such tests for anti-viral antibodies can only be detected several days after infection onset, and can persist even after clearance of the virus, when a patient is no longer contagious.^2^ As such, the presence of antibodies detected in such home-kits may only provide an indirect indication of previous viral exposure rather than reporting on actual viral load, a critical parameter for minimizing the spread of SARS-CoV-2.

At present, the gold standard detection method of viral nucleic acids in patients is performed in laboratories by professionals. As opposed to antibody testing, detection of viral RNA offers a direct measure of a patient’s contagiousness. At this point in the current COVID-19 pandemic, it is clear that the availability and throughput of standard methods for viral nucleic acid detection are limited both in terms of resources, and accessibility to the community.

**Figure 1.**
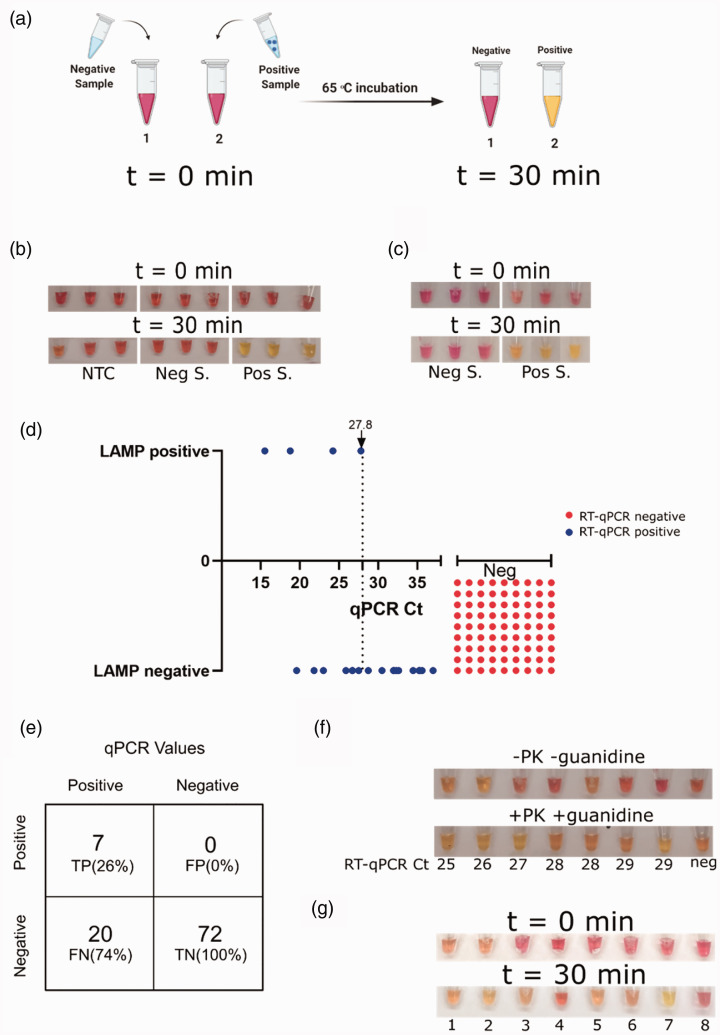
Protocol adjustment and optimal conditions. (a) Schematic representation of the isothermal colorimetric RT-LAMP reaction. (b) RT-LAMP reaction performed on purified RNA from nasal and throat swabs submerged in UTM buffer. Results from a no-template control (NTC; left), a negative subject (Neg S.; middle), and a positive subject (Pos S.; right) at t = 0 (upper panels) and after a 30-min incubation at 65°C (lower panels) are shown. Three technical replicates of each sample are shown. (c) Representative RT-LAMP test results of clinical diagnostic nasal and throat swabs. Three different negative samples (Neg S.; left), and three different positive samples (Pos S.; right) obtained at t = 0 and t = 30 min were directly tested with no RNA purification step. (d) Comparison of the RT-LAMP method to Ct values obtained by standard RT-qPCR (3 true positive and 2 false negative samples of the 99 samples analyzed are not shown due to inaccessibility to their RT-qPCR Ct values). RT-qPCR negative samples were assigned arbitrary Ct values, for visualization. (e) Classification of true positive (TP), true negative (TN), false positive (FP) and false negative (FN) numbers and rate of RT-LAMP test results, as compared to the standard RT-qPCR test results. (f) Clinical diagnostic nasal and throat swabs tested by two different RT-LAMP protocols. Upper panel, without proteinase K and guanidine hydrochloride. Lower panel, with proteinase K and guanidine hydrochloride. For RT-qPCR positive samples, the Ct value is presented under each sample. The sample to the right is negative. (g) RT-LAMP results for samples from patients confirmed as positive for the following viruses, with the fraction tested indicated: 1–2, HSV; swabs (lysed and inactivated as described in the currently developed protocol). 3, HSV; purified DNA. 4, RSV; purified RNA. 5, Influenza B; RNA. 6, Enterovirus; RNA. 7, RNA extraction from a SARS-CoV-2 positive patient. 8, no template control. Results are shown at t = 0 and 30 min after incubation at 65 ͦC.

Standard methods used for detecting viral RNA in patients include RNA purification, reverse transcription, and quantitative PCR (RT-qPCR). These processes are time-consuming, require multiple biochemical reagents, lab-grade instruments, and trained professionals.^3^ Fortunately, alternative molecular biology methods can overcome these limitations. Colorimetric loop-mediated isothermal amplification (LAMP) is one such method.^4^ Reverse-transcribed (RT)-LAMP is performed at a single, constant temperature for one-step reverse transcription and genomic material amplification. This reaction is based on three pairs of primers that produce stem-loop DNA structures, which serve as templates for exponential amplifications. RT-LAMP provides results that can be visualized by color change, thereby eliminating the need for sophisticated lab equipment other than a constant heat source (for graphical presentation, see Figure 1(a)).^5–7^ The colorimetric detection in this method is based on the pH-sensitive dye—phenol red. Incorporation of dNTPs during DNA amplification results in accumulation of protons that reduces the pH in the reaction buffer and can be detected by a color change. In acidic pH, phenol red changes color to yellow.^8^ Here, we adjusted RT-LAMP for the detection of SARS-CoV-2 RNA directly from clinical diagnostic swabs sampled from human patients, without any RNA purification steps. To evaluate the validity of our method, we compared our results to those obtained by the approved testing method at the Rambam Health Care Campus (RHCC) hospital.

## Materials and methods

### Samples collection

Patient throat and nose swabs were collected and transferred to one tube by field healthcare workers and sent to the Virology laboratory at the Rambam Health Care Campus, Haifa, Israel. The swabs were stored in 1–2 mL universal transfer media (UTM). Saliva samples were self-collected directly into sterile cups and kept at 4°C until tested.

### RT-qPCR

Viral RNA was extracted using one of three automated nucleic acid extraction systems (easyMAG/EMAG (Biomeriuex), magLEAD 5bL (Precision System Science) or MagEx (STARlet)), from a mixture containing 2 mL lysis buffer, 0.5 mL sample and 50 µL elution buffer or 270 µL lysis buffer, 130 µL sample and 50 µL elution buffer or 300 µL lysis buffer, 400 µL sample and 50 µL elution buffer, respectively. Following viral RNA extraction, RT-qPCR was performed using one of two commercial kits (Allplex 2019-nCoV (Seegene) or real-time fluorescent RT-PCR Kit for Detecting SARS-2019-nCoV (BGI)), according to the manufacturer’s instructions. Additional RT-qPCR reaction mix was created manually using custom-made primers (see Table 1).^4,9^ Probe IC was synthesized to present a 5'-FAM/CY5 fluorophore moiety and a 3'-ZEN/IBFQ quencher. Each of the manual reactions contained 2X Ag-Path One-step mix (12.5 µL; Ambion), primers (1 µL), reverse transcriptase (1 µL; SOURCE), previously extracted sample RNA (5 µL), and H_2_O to a final volume of 25 µL. All RT-qPCR reactions were executed in either a Quantstudio (Thermo Fisher Scientific) or CFX96 (Bio-Rad) RealTime PCR machine under the following conditions: 30 min at 50°C, 10 min at 95°C, and 45 cycles of a two-step incubations comprising a 15 s step at 95°C and a 30 s step at 55°C. Fluorescence was measured during step 2 of each cycle.

**Table 1.** Primers used in this study.

Primer Name	Sequence	Final conc. [nM]
RT-qPCR primers		
E_Sarbeco_R2	ATATTGCAGCAGTACGCACACA	400
E_Sarbeco_P1	ACACTAGCCATCCTTACTGCGCTTCG	200
E_Sarbeco_F1	ACAGGTACGTTAATAGTTAATAGCGT	400
Primer FW IC (Upstream/1/Fw)	CATGGGAAGCAAGGGAACTAATG	250
Primer RV IC (Downstream/2/Rv)	CCCAGCGAGCAATACAGAATTT	250
Probe IC	5’ –CY5–TCTTCCCTCGAACCTGCACCATCAAT-3′	250
RT-LAMP primers		
**Primer Name**	**Sequence**	**Final conc. [nM]**
GeneN-A-F3	TGG CTA CTA CCG AAG AGC T	200
GeneN-A-B3	TGC AGC ATT GTT AGC AGG AT	200
GeneN-A-LF (Loop Forward)	GGA CTG AGA TCT TTC ATT TTA CCG T	400
GeneN-A-LB (Loop Backward)	ACT GAG GGA GCC TTG AAT ACA	400
GeneN-A-FIP (Forward Inner Primer)	TCT GGC CCA GTT CCT AGG TAG TCC AGA CGA ATT CGT GGT GG	1600
GeneN-A-BIP (Backward Inner Primer)	AGA CGG CAT CAT ATG GGT TGC ACG GGT GCC AAT GTG ATC T	1600
RNaseP POP7 F3	TTGATGAGCTGGAGCCA	200
RNaseP POP7 B3	CACCCTCAATGCAGAGTC	200
RNaseP POP7 LF	ATGTGGATGGCTGAGTTGTT	400
RNaseP POP7 LB	CATGCTGAGTACTGGACCTC	400
RNaseP POP7 FIP	GTGTGACCCTGAAGACTCGGTTTTAGCCACTGACTCGGATC	1600
RNaseP POP7 BIP	CCTCCGTGATATGGCTCTTCGTTTTTTTCTTACATGGCTCTGGTC	1600

Note: GeneN-A primers were described by Zhang *et al*.^4^ RNase P POP7 primers were described by Curtis *et al*.^9^

### Colorimetric RT-LAMP reaction

Samples in UTM (5 µL) were diluted in 40 µL DNase RNase free water (Biological Industries, 01–869-1B) containing 2 µL of Proteinase K (final concentration, 1.22 mg/mL; Seegene, 744300.4.UC384) and incubated at room temperature for 15 min. Proteinase K was inactivated by incubating the reaction tubes at 95°C for 5 min. Next, a colorimetric RT-LAMP reaction was performed in a total volume of 20 µL per reaction using 10 µL WarmStart Colorimetric LAMP 2X Master Mix (New England BioLabs, M1800), 2 µL primers mix (see Table 1), 1 µL guanidine hydrochloride (final concentration of 40 mM; Sigma, G4505), and 7 µL of the inactivated sample. For calibration, reactions were performed with or without proteinase K and/or guanidine hydrochloride. The reaction was then incubated for 30–40 min at 65°C. The proteinase K inactivation and reaction steps were performed in a closed-lid heating block or, for proof of concept, in a Thermos cup, using warm water. In the case of the Thermos cup, temperature was monitored using a simple thermometer. After 20 min of incubation, aliquots were monitored for color change every 5 min using the naked eye, until 40 min of incubation. Samples were considered negative if the original pink color of the phenol red was maintained and positive if the phenol red color turned orange yellow. Test results were interpreted without prior knowledge of the reference standard results.

### Statistical analysis

True positive rate (TPR), true positive rate (TNR), true negative rate (FPR), false positive rate (FNR), and false negative rate (FNR) values were calculated according to the following equations: TPR = TP/(TP+FN). TNR = TN/(FP+TN). FNR = FN/(TP+FN). FPR = FP/(FP+TN).

## Results

### Optimal conditions for colorimetric SARS-CoV-2 detection by RT-LAMP

We first performed RT-LAMP on RNA purified from COVID-19-positive and -negative swabs, with the set of primers described by Zhang *et al.*^4^ As reported by Zhang *et al.*,^4^ our RT-LAMP results agreed with those obtained using standard RT-qPCR (Figure 1(b)). To simplify the detection method, we next conducted RT-LAMP on clinical diagnostic throat and nose swabs collected from patients and maintained in UTM. After inactivating the samples by heating the UTM to 95°C for 5 min, the inactivated samples from confirmed patients were deemed as positive by the RT-LAMP reaction (Figure 1(c)). We then evaluated this RT-LAMP protocol on a cohort of 99 patients that were previously tested in hospital labs by the standard RT-qPCR method. The tested pool included 27 positive samples containing a wide range of viral load and 72 negative samples. The detection limit of the RT-LAMP protocol was at an RT-qPCR cycle threshold (Ct) of 27.8 (Figure 1(d), Supplementary Table 1), with 7 true positives (TP), 20 false negatives (FN), 72 true negatives (TN), and no false positives (FP) (Figure 1(e), Supplementary Table 1). Although no FPs were noted, the number of TPs was very low. Hence, we looked to improve the RT-LAMP protocol for detection from clinical diagnostic patient swabs. Since these swab samples may contain enzymatic inhibitors that could affect the efficiency of viral RNA detection, the impacts of adding proteinase K and guanidine hydrochloride to the process were tested. Proteinase K was added to UTM samples, while guanidine hydrochloride was introduced at the RT-LAMP reaction step. We compared the two protocols on samples from eight patients presenting low, medium, and high Ct values. Of seven patients deemed positive by RT-qPCR (Ct < 37), two were clearly RT-LAMP-positive according to the former protocol, while two additional ones (i.e. a total of four) were identified as positive using the modified protocol. We concluded that the adjustments made improved the efficiency of the RT-LAMP protocol to directly detect viral RNA in clinical diagnostic swab samples (Figure 1(f)). Lastly, we also tested samples from patients confirmed as infected with viruses other than SARS-CoV-2, including Herpes simplex virus (HSV), respiratory syncytial virus (RSV), influenza and enterovirus. As shown in Figure 1(g), our RT-LAMP protocol deemed these individuals as negative, and only the sample containing SARS-CoV-2 RNA has tested positive.

### Applying RT-LAMP to a cohort of 83 suspected SARS-CoV2 patients

**Figure 2.**
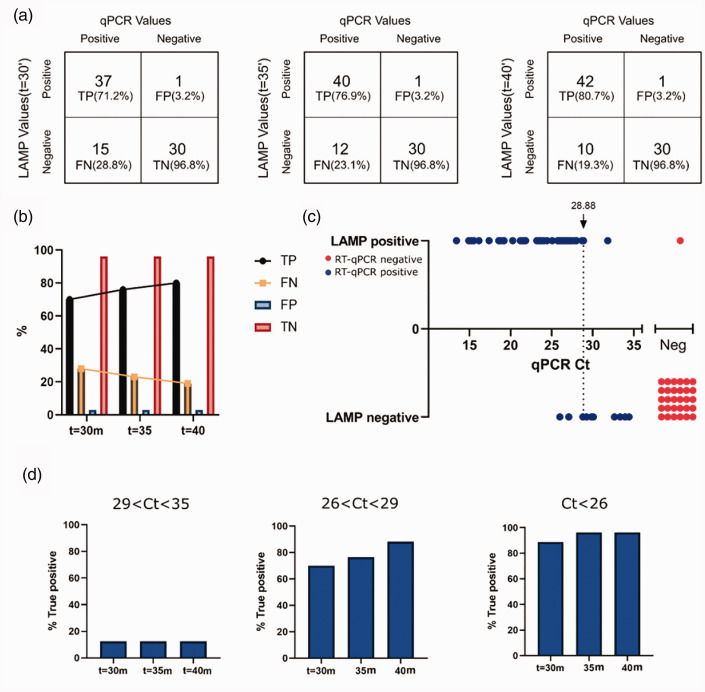
Adjusted RT-LAMP protocol used to test 83 clinical diagnostic nasal and throat swab samples. (a) Distribution of TP, TN, FP, and FN readings and rates of RT-LAMP test results, as compared to standard RT-qPCR results. Boxes from left to right represent results at t = 30, 35, and 40 min, respectively. (b) Bar graph representation of TP, TN, FP, and FN rates shown in (a). (c) Comparison of the RT-LAMP method to Ct values obtained with standard RT-qPCR. (d) Graphical representation of TP rates of RT-LAMP after incubations of 30 min, 35 min and 40 min, compared to RT-qPCR test results separated by Ct value intervals. 29<Ct<35 (left), 26<Ct<29, (middle), Ct<26 (right).

With this adjusted protocol, we next tested its validity on a cohort of 83 patients suspected of being infected with SARS-CoV-2. These patients were tested at RHCC using the standard RT-qPCR method, 31 were deemed negative and 52 were recognized as positive, presenting a wide range of RT-qPCR Ct values (14–35). To determine the optimal incubation time needed to yield the highest number of true positives without increasing the rate of false negatives, we performed RT-LAMP for up to 40 min and evaluated the colorimetric results obtained at the 30, 35, and 40-min time points. We thus found that the number of TPs increased, while the number of FNs decreased over time. No change in the number of TNs was noted, and only one FP was reported throughout (Figure 2(a) to (c), Supplementary Table 2). Readings collected after 35 and 40 min showed the highest TP rate in samples presenting low (Ct < 26) and medium (26<Ct < 29) Ct values (Figure 2(d), Supplementary Table 2). Hence, in the conditions employed, it was concluded that incubation times of 35 and 40 min were optimal. Results obtained after a 40-min incubation are provided in Figure 2(c). When these results were compared to RT-qPCR Ct values obtained from the same patient samples, it was determined that RT-LAMP was most sensitive in terms of detecting positive patients with viral loads that corresponds to low and medium RT-qPCR Ct values. For Ct values under 28.8, the TP rate reported by RT-LAMP was 93%.

### Applying RT-LAMP protocol on saliva samples from confirmed patients

**Figure 3.**
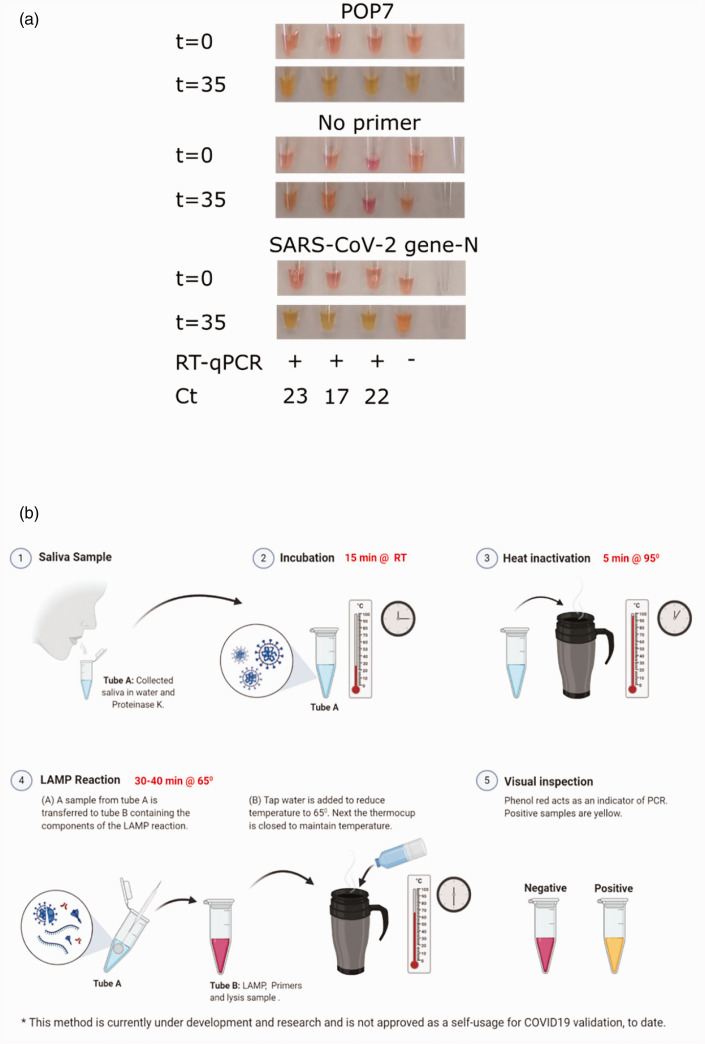
Employing the RT-LAMP protocol with saliva samples. (a) RT-LAMP tests were performed on saliva from four volunteers. Each tube represents one tested volunteer. Results attained at t = 0 and t = 35 are shown. Upper panel, RT-LAMP reaction using *pop7* primers as a positive control. Middle panel, control for RT-LAMP reaction with no primers. Lower panel, RT-LAMP reaction using SARS-CoV-2 gene N primers. The same samples were analyzed with the conventional hospital RT-qPCR protocol. The RT-qPCR results and Ct values are shown under the relevant samples. (b) Graphical illustration of the potential of RT-LAMP protocol for saliva self-testing.

Human to human transmission of SARS-CoV-2 is mainly mediated by saliva droplets.^10^ Comparing saliva samples to the standard swabs revealed the presence of higher viral loads in saliva.^11^ Indeed, the FDA has recently approved saliva as a source for detecting COVID-19 (https://www.fda.gov/media/136875/download). Therefore, we next performed RT-LAMP on human saliva samples self-collected from three different COVID-19-confirmed patients and one suspected negative subject. In parallel, we also performed the standard RNA purification and RT-qPCR reactions on the saliva samples in a hospital setting. Testing saliva samples from the confirmed patients by both RT-LAMP and RT-qPCR confirmed the positive assessments, whereas the suspected negative subject was confirmed as being negative (Figure 3(a)). As a positive control for the reaction and saliva sampling, we tested for the human *pop7* gene in addition to SARS-CoV-2 gene N. Indeed, *pop7* was detected in all saliva samples (Figure 3(a)).

## Discussion

RT-LAMP is a rapid and simple method to detect purified SARS-CoV-2 RNA.^4^ In this study, we addressed the potential of RT-LAMP as a method for the direct detection of the virus in suspected COVID-19 patients. Accordingly, we modified and validated the RT-LAMP protocol for direct SARS-CoV-2 detection from clinical diagnostic patient swab samples. Our experiments were performed in parallel to the standard RT-qPCR, hospital routine method. We thus compared RT-qPCR Ct values to RT-LAMP results from over 180 different patient samples. Upon calibration, our direct RT-LAMP method successfully detected patients with medium to high viral loads, while yielding very few false positives.

In this work, we defined the optimal protocol for immediate off-the-shelf use of RT-LAMP in testing putative COVID-19 patients. Most importantly, our protocol does not include an RNA purification step. In fact, other than a constant heat source (e.g. a thermal mug), no sophisticated equipment is required in this approach. The protocol duration is about an hour from sampling to detection, calls for only few reagents, and can potentially be performed by non-professionals or even self-performed (Figure 3(b)). These features allow for the implementation of our method around the globe, including rural areas.

Here we focused on clinical swabs and a few self-collected saliva samples. To further encourage use of RT-LAMP as a method for identifying COVID-19 patients, we suggest testing this protocol on a larger cohort of human saliva samples. Upon such further validation, this method of SARS-CoV-2 detection can be employed as a surveillance tool for sampling large populations. Indeed, the simplicity of this method, the ready availability of products and its low cost, together will make it easy to continuously monitor suspected infected individuals. This method can, moreover, be used in numerous settings, including medical clinics, nursing homes, the workplace, and at points of entry. Finally, and no less important, this method can easily be adjusted to test for infection by other pathogens.

## Supplemental Material

Supplemental_Material.pdf - Supplemental material for Direct on-the-spot detection of SARS-CoV-2 in patients Click here for additional data file. Supplemental material, sj-zip-1-ebm-10.1177_1535370220941819 for Direct on-the-spot detection of SARS-CoV-2 in patients by Nadav Ben-Assa, Rawi Naddaf, Tal Gefen, Tal Capucha, Haitham Hajjo, Noa Mandelbaum, Lilach Elbaum, Peter Rogov, Daniel A King, Shai Kaplan, Assaf Rotem, Michal Chowers, Moran Szwarcwort-Cohen, Mical Paul and Naama Geva-Zatorsky in Experimental Biology and Medicine
