# Supplementary material for: Direct on-the-spot detection of SARS-CoV-2 in patients
Source: Exp Biol Med (Maywood). 2020 Jul 16;245(14):1187–93. doi: 10.1177/1535370220941819 (PMC7385438; doi:10.1177/1535370220941819)
Supplement: Supplemental_Material.pdf - Supplemental material for Direct on-the-spot detection of SARS-CoV-2 in patients [file Supplemental_Material.pdf]

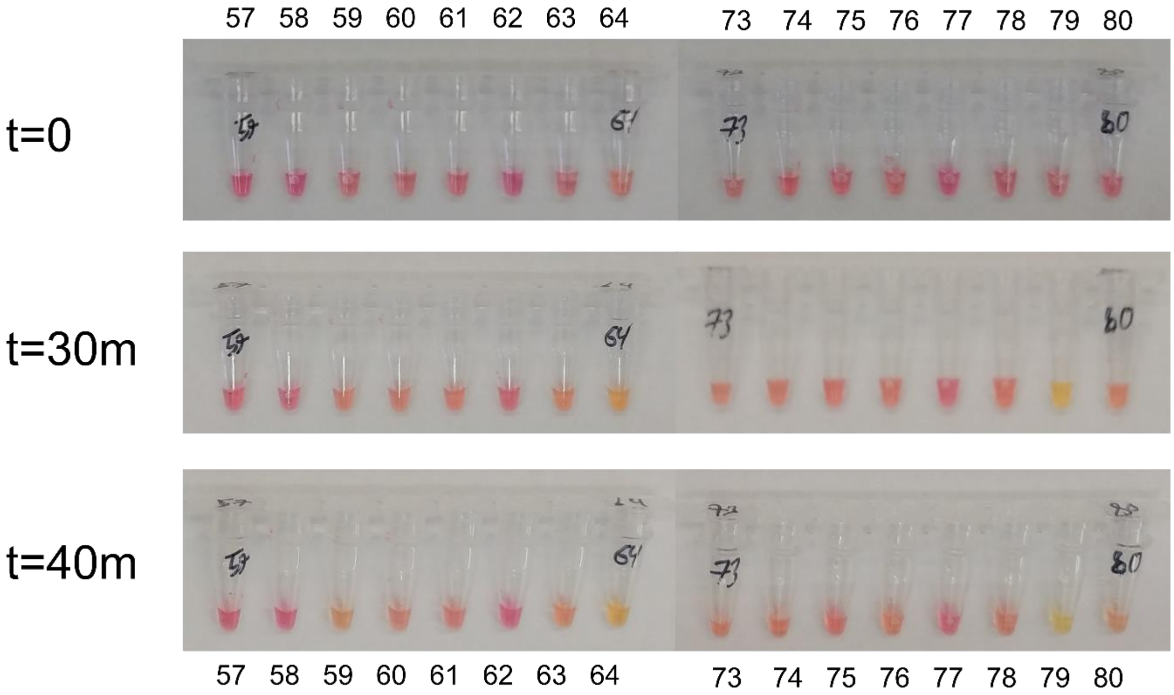

Supplementary figure 1:

Representative pictures of data related to Figure 1d,e and Supp. Table 1. RT-LAMP reaction products at t = 0, 30 and 40 minutes are shown. Numbers above and under each tube corresponds to the number of the sample in Supp. Table 1.

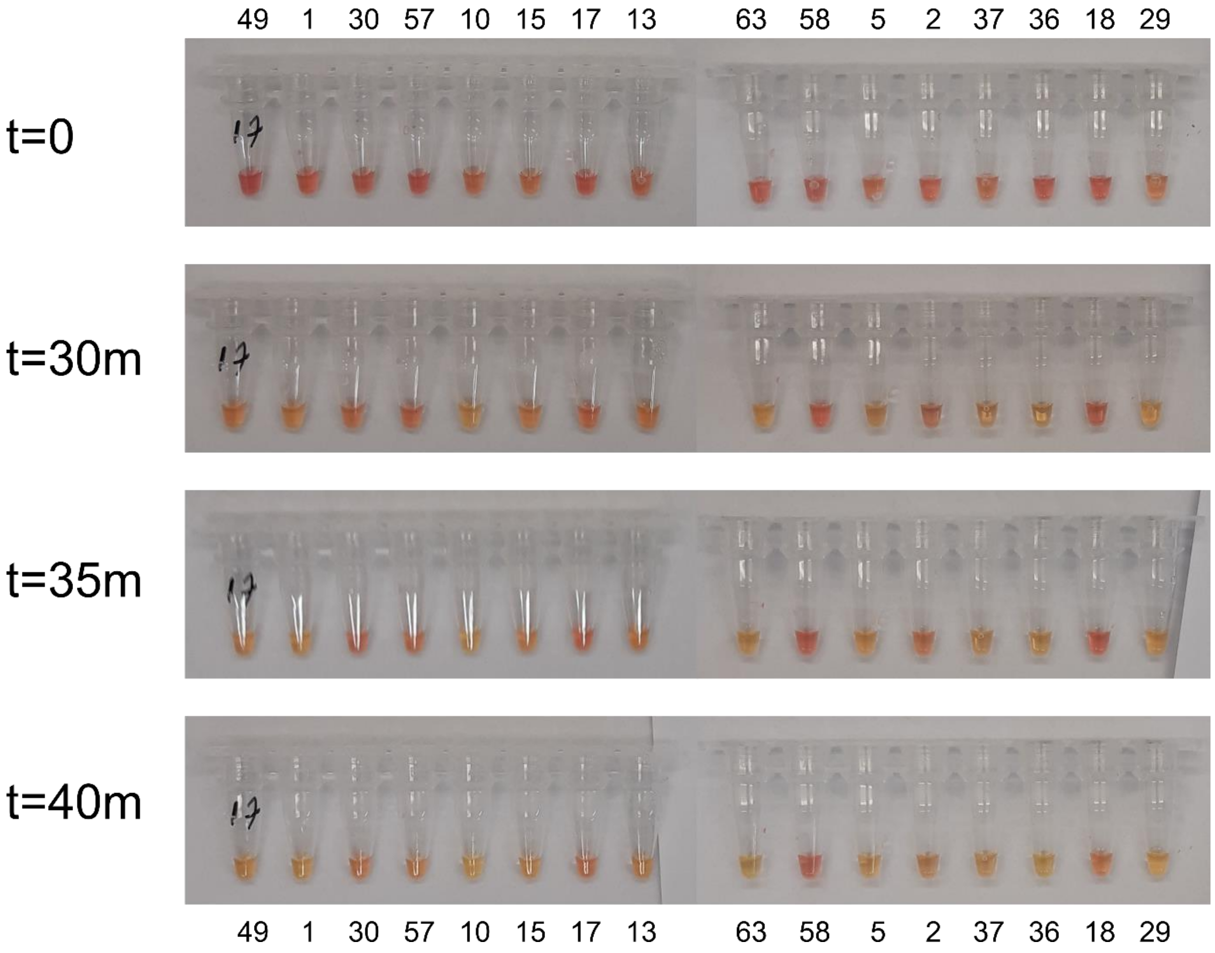

Supplementary figure 2:

Representative pictures of data presented in Figure 2 and Supp. Table 2. RT-LAMP reaction products at t = 30, 35 and 40 minutes are shown. Numbers above and under each tube corresponds to the number of the sample in Supp. Table 2.

Supplementary Table 1:

Sample#	Sample type	Ct	RT-PCR result	Swab buffer	Test results t(30) min (0-negative, 1-positive)	Test results t(40) min (0-negative, 1-positive)
1	Swab	-	0	Serological tube red cup Pink buffer	0	0
2	Swab	-	0	Serological tube red cup Pink buffer	0	0
3	Swab	-	0	Serological tube red cup Pink buffer	0	0
4	Swab	-	0	Serological tube red cup Pink buffer	0	0
5	Swab	-	0	Serological tube red cup Pink buffer	0	0
6	Swab	-	0	Serological tube red cup Pink buffer	0	0
7	Swab	-	0	Serological tube red cup Pink buffer	0	0
8	Swab	-	0	Serological tube red cup Pink buffer	0	0
9	Swab	-	0	Serological tube red cup Pink buffer	0	0
10	Swab	-	0	Serological tube red cup Pink buffer	0	0
11	Swab	-	0	Serological tube red cup Pink buffer	0	0
12	Swab	-	0	Serological tube red cup Pink buffer	0	0
13	Swab	-	0	Serological tube red cup Pink buffer	0	0
14	Swab	-	0	Serological tube red cup Pink buffer	0	0
15	Swab	-	0	Serological tube red cup Pink buffer	0	0
16	Swab	-	0	Serological tube red cup Pink buffer	0	0
17	Swab	-	0	Serological tube red cup Pink buffer	0	0
18	Swab	-	0	Serological tube red cup Pink buffer	0	0
19	Swab	-	0	Serological tube red cup Pink buffer	0	0
20	Swab	-	0	Serological tube red cup Pink buffer	0	0
21	Swab	-	0	Serological tube red cup Pink buffer	0	0
22	Swab	-	0	Serological tube red cup Pink buffer	0	0
23	Swab	-	0	Serological tube red cup Pink buffer	0	0
24	Swab	-	0	Serological tube red cup Pink buffer	0	0
25	Swab	-	0	Serological tube red cup Pink buffer	0	0
26	Swab	-	0	Serological tube red cup Pink buffer	0	0
27	Swab	-	0	Serological tube red cup Pink buffer	0	0
28	Swab	-	0	Serological tube red cup Pink buffer	0	0
29	Swab	-	0	Serological tube red cup Pink buffer	0	0
30	Swab	-	0	Serological tube red cup Pink buffer	0	0
31	Swab	-	0	Serological tube red cup Pink buffer	0	0
32	Swab	-	0	Serological tube red cup Pink buffer	0	0
33	Swab	-	0	Serological tube red cup Pink buffer	0	0
34	Swab	-	0	Serological tube red cup Pink buffer	0	0
35	Swab	-	0	Serological tube red cup Pink buffer	0	0
36	Swab	-	0	Serological tube red cup Pink buffer	0	0
37	Swab	-	0	Serological tube red cup Pink buffer	0	0
38	Swab	-	0	Serological tube red cup Pink buffer	0	0
39	Swab	-	0	Serological tube red cup Pink buffer	0	0

40	Swab	-	0	Serological tube red cup Pink buffer	0	0
41	Swab	-	0	Serological tube red cup Pink buffer	0	0
42	Swab	-	0	Serological tube red cup Pink buffer	0	0
43	Swab	-	0	Serological tube red cup Pink buffer	0	0
44	Swab	-	0	Serological tube red cup Pink buffer	0	0
45	Swab	-	0	Serological tube red cup Pink buffer	0	0
46	Swab	-	0	Serological tube red cup Pink buffer	0	0
47	Swab	-	0	Serological tube red cup Pink buffer	0	0
48	Swab	-	0	Serological tube red cup Pink buffer	0	0
49	Swab	-	0	Serological tube red cup Pink buffer	0	0
50	Swab	-	0	Serological tube red cup Pink buffer	0	0
51	Swab	-	0	Serological tube red cup Pink buffer	0	0
52	Swab	-	0	Serological tube red cup Pink buffer	0	0
53	Swab	-	0	Serological tube red cup Pink buffer	0	0
54	Swab	-	0	Serological tube red cup Pink buffer	0	0
55	Swab	-	0	Serological tube red cup Pink buffer	0	0
56	Swab	-	0	Serological tube red cup Pink buffer	0	0
57	Swab	-	0	Serological tube red cup Pink buffer	0	0
58	Swab	-	0	Serological tube red cup Pink buffer	0	0
59	Swab	-	0	15 ml falcon orenge cup clear buffer	0	0
60	Swab	-	0	15 ml falcon orenge cup clear buffer	0	0
61	Swab	-	0	15 ml falcon orenge cup clear buffer	0	0
62	Swab	-	0	15 ml falcon orenge cup clear buffer	0	0
63	Swab	-	0	Serological tube red cup Pink buffer	0	0
64	Swab	+	1	Serological tube blue cup yellow buffer	1	1
65	Swab	+	1	Serological tube red cup Pink buffer	1	1
66	Swab	35.22	1	Serological tube blue cup yellow buffer	0	0
67	Swab	-	0	Falcon 15 ml Blue cap redish buffer	0	0
68	Swab	25.87	1	Falcon 15 ml Blue cap redish buffer	0	0
69	Swab	18.76	1	Falcon 15 ml Blue cap redish buffer	1	1
70	Swab	-	0	Falcon 15 ml Blue cap redish buffer	0	0
71	Swab	+	1	15 ml falcon orenge cup clear buffer	0	0
72	Swab	+	1	15 ml falcon orenge cup clear buffer	1	1
73	Swab	+	1	Falcon 15 ml Blue cap redish buffer	0	0
74	Swab	34.46	1	Falcon 15 ml Blue cap redish buffer	0	0
75	Swab	32.31	1	15 ml falcon orenge cup clear buffer	0	0
76	Swab	27.51	1	Falcon 15 ml Blue cap redish buffer	0	0
77	Swab	19.58	1	Serological tube red cup Pink buffer	0	0
78	Swab	-	0	Serological tube red cup Pink buffer	0	0
79	Swab	15.51	1	15 ml falcon orenge cup clear buffer	1	1
80	Swab	-	0	15 ml falcon orenge cup clear buffer	0	0
81	Swab	24.22	1	Serological tube red cup Pink buffer	1	1
82	Swab	30.47	1	15 ml falcon orenge cup clear buffer	0	0
83	Swab	21.8	1	Serological tube red cup Pink buffer	0	0

84	Swab	23	1	Serological tube red cup Pink buffer	0	0
85	Swab	27.8	1	Serological tube red cup Pink buffer	1	1
86	Swab	37	1	Serological tube red cup Pink buffer	0	0
87	Swab	32	1	Serological tube red cup Pink buffer	0	0
88	Swab	26.72	1	Serological tube red cup Pink buffer	0	0
89	Swab	-	0	Serological tube red cup Pink buffer	0	0
90	Swab	35.6	1	Falcon 15 ml Blue cap redish buffer	0	0
91	Swab	32.6	1	Serological tube red cup Pink buffer	0	0
92	Swab	31.95	1	Serological tube red cup Pink buffer	0	0
93	Swab	-	0	Serological tube red cup Pink buffer	0	0
94	Swab	-	0	Serological tube red cup Pink buffer	0	0
95	Swab	-	0	Serological tube red cup Pink buffer	0	0
96	Swab	35.3	1	Serological tube red cup Pink buffer	0	0
97	Swab	26.7	1	Serological tube red cup Pink buffer	0	0
98	Swab	28.72	1	Serological tube red cup Pink buffer	0	0
99	Swab	-	0	Falcon 15 ml Blue cap redish buffer	0	0

Supplementary Table 2:

Sample#	Sample type	CT	RT-PCR result	Test results t(30) min (0-negative, 1-positive)	Test results t(35) min (0-negative, 1-positive)	Test results t(40) min (0-negative, 1-positive)
1	swab	26.48	1	1	1	1
2	swab	28.02	1	1	1	1
3	swab	30.07	1	0	0	0
4	swab	32.64	1	0	0	0
5	swab	19.2	1	1	1	1
6	swab	23.18	1	1	1	1
7	swab	28.7	1	1	1	1
8	swab	17.38	1	1	1	1
9	swab	25.06	1	0	1	1
10	swab	24.43	1	1	1	1
11	swab	14.9	1	1	1	1
12	swab	28.88	1	1	1	1
13	swab	27.26	1	1	1	1
14	swab	13.4	1	1	1	1
15	swab	27.03	1	0	1	1
16	swab	23.71	1	1	1	1
17	swab	27.08	1	0	0	0
18	swab	21.19	1	1	1	1
19	swab	18.77	1	1	1	1
20	swab	21.6	1	1	1	1
21	swab	20.25	1	1	1	1
22	swab	26.49	1	1	1	1
23	swab	25.87	1	0	1	1
24	swab	25.84	1	1	1	1
25	swab	15.26	1	1	1	1
26	swab	15.51	1	1	1	1
27	swab	21.44	1	1	1	1
28	swab	23.6	1	1	1	1
29	swab	15.22	1	1	1	1
30	swab	26.77	1	0	0	1
31	swab	27	1	1	1	1
32	swab	16.15	1	1	1	1
33	swab	27.57	1	1	1	1
34	swab	25.72	1	1	1	1
35	swab	24.03	1	1	1	1
36	swab	25.99	1	0	0	0
37	swab	26.01	1	1	1	1

38	swab	26.29	1	1	1	1
39	swab	23.49	1	1	1	1
40	swab	29.25	1	0	0	0
41	swab	29.84	1	0	0	0
42	swab	19.1	1	1	1	1
43	swab	28.81	1	0	0	0
44	swab	26.04	1	1	1	1
45	swab	18.55	1	1	1	1
46	swab	34.41	1	0	0	0
47	swab	33.96	1	0	0	0
48	swab	21.82	1	1	1	1
49	swab	31.82	1	1	1	1
50	swab	33.34	1	0	0	0
51	swab	27.91	1	1	1	1
57	swab	26.72	1	0	0	1
52	swab	-	0	0	0	0
53	swab	-	0	0	0	0
54	swab	-	0	0	0	0
55	swab	-	0	1	1	1
56	swab	-	0	0	0	0
58	swab	-	0	0	0	0
59	swab	-	0	0	0	0
60	swab	-	0	0	0	0
61	swab	-	0	0	0	0
62	swab	-	0	0	0	0
63	swab	-	0	0	0	0
64	swab	-	0	0	0	0
65	swab	-	0	0	0	0
66	swab	-	0	0	0	0
67	swab	-	0	0	0	0
68	swab	-	0	0	0	0
69	swab	-	0	0	0	0
70	swab	-	0	0	0	0
71	swab	-	0	0	0	0
72	swab	-	0	0	0	0
73	swab	-	0	0	0	0
74	swab	-	0	0	0	0
75	swab	-	0	0	0	0
76	swab	-	0	0	0	0
77	swab	-	0	0	0	0
78	swab	-	0	0	0	0
79	swab	-	0	0	0	0
80	swab	-	0	0	0	0
81	swab	-	0	0	0	0
82	swab	-	0	0	0	0
83	swab	-	0	0	0	0
